# IMPACT OF RECEIVING HOME-BASED PRIMARY CARE FOR PERSONS LIVING WITH DEMENTIA: PATIENT AND CAREGIVER PERSPECTIVES

**DOI:** 10.1093/geroni/igac059.3027

**Published:** 2022-12-20

**Authors:** Meghan Mattos, Veronica Bernacchi, Karen Duffy, Laura Jepson, Stephanie Gonzalez, Justin Mutter

**Affiliations:** University of Virginia, School of Nursing, Charlottesville, Virginia, United States; Penn State College of Medicine, State College, Pennsylvania, United States; UVA Health, Charlottesville, Virginia, United States; University of Virginia, Charlottesville, Virginia, United States; University of Virginia, School of Nursing, Charlottesville, Virginia, United States; University of Virginia, Charlottesville, Virginia, United States

## Abstract

Homebound persons living with dementia (PWD) require healthcare services that proliferate with physical and cognitive changes over time. PWD often experience fragmented care that results in poor health outcomes and delayed healthcare access. The objective of the Virginia at Home Program (VaH), a recently initiated home-based primary care (HBPC) program, is to promote long-term sustainability and proliferation of HBPC. The study purpose was to examine the impact of VaH on the overall care and outcomes of homebound PWD and their families. We used quantitative and qualitative methods to describe the impact of VaH on patient care and outcomes over six months. We compared clinical outcomes (e.g., activities of daily living, frailty, behavioral symptoms) at baseline and six months using paired t-tests. We also conducted semi-structured phone interviews at baseline and six months with PWD-caregiver dyads. Thirty PWD-caregiver dyads enrolled. Of the 17 dyads that completed both visits, PWD’s physical function and caregiver’s burden decreased significantly over time (ps < .05). There was a significant improvement in PWD’s behavioral symptoms over the six-month period (p=.03). Three preliminary themes emerged from qualitative data: 1) participants established trusting relationships with the VaH team, 2) VaH supported caregivers in their caregiving responsibilities, and 3) participants met their pre-program goals through increased healthcare access. Findings suggest that the HBPC intervention improved care satisfaction and healthcare access and alleviated caregiver burden. Future studies should consider introducing HBPC programs in this hard-to-reach population to decrease acute care utilization and health care costs while improving care satisfaction and quality.

